# Field-based evaluation of novaluron EC10 insect growth regulator, a chitin synthesis inhibitor against dengue vector breeding in leaf axils of pineapple plantations in Gampaha District, Sri Lanka

**DOI:** 10.1186/s13071-020-04109-y

**Published:** 2020-05-06

**Authors:** Nayana Gunathilaka, Tharaka Ranathunga, Dakshina Hettiarachchi, Lahiru Udayanga, Wimaladharma Abeyewickreme

**Affiliations:** 1 Department of Parasitology, Faculty of Medicine, University of Kelaniya, Ragama, Sri Lanka; 2 Molecular Medicine Unit, Faculty of Medicine, University of Kelaniya, Ragama, Sri Lanka; 3 A. Baurs Co. Ltd, Colombo, Sri Lanka; 4 Department of Bio-systems Engineering, Faculty of Agriculture and Plantation Management, Wayamba University of Sri Lanka, Makandura, Sri Lanka

**Keywords:** Insect growth regulator, *Aedes*, Field efficacy, Pineapple plantation, Novaluron

## Abstract

**Background:**

Insect growth regulators (IGRs) are considered a novel group of insecticides to control mosquitoes. Novaluron is an IGR with benzoylphenyl urea insecticide, which inhibits chitin synthesis in insects and can reduce insect population density; it is also known to have a high margin of safety for mammals.

**Methods:**

The effective minimum concentration of novaluron formulation EC10 was tested. Six pineapple plantations [control (*n* = 3) and test (*n* = 3)] were selected from Meerigama Medical Officer of Health area in Gampaha District, Sri Lanka. Fifteen plots (10 × 10 m) were demarcated in each site with a 200 m distance apart. Leaf axils of 450 pineapple plants (30 plants × 15 plots) were screened for immature stages of *Aedes* mosquitoes weekly for 12 weeks. The required concentration (20 ppm) of novaluron was sprayed onto the selected pineapple plants (*n* = 1350) individually in 3 selected test sites for 5–10 s. The reduction in the vector population was interpreted as the percentage of reduction in immature stages of *Aedes* mosquitoes.

**Results:**

The 100% mortality of the *Ae. aegypti* larvae within 24 h was observed at 20 ppm (0.05 ml of novaluron 100 g/l in 250 ml of water) as the minimum dose. Variation in the number of *Aedes* larvae present in the control and intervention sites was found to be significantly different throughout the entire observational period (*χ*^2^ = 128.29, *df* = 11, *P *< 0.001). The total elimination of *Aedes* larvae continued for up to 2 weeks and a 50% reduction was observed until the 8th week.

**Conclusions:**

The present study emphasizes that novaluron (10% EC) can be used as an effective larvicide at the treatment dose of 20 ppm. The residual effect of the IGR lasted for 12 weeks with a functional efficacy of 8 weeks. The 100% reduction of larval breeding was observed up to the 2nd week after application and the percentage reduction of immature stages remained > 50% until the 8th week. The lowest reduction (34.2%) was observed at 12 weeks after the initial treatment. Therefore, re-treatment may be recommended based on the reduction in the efficacy of the IGR.
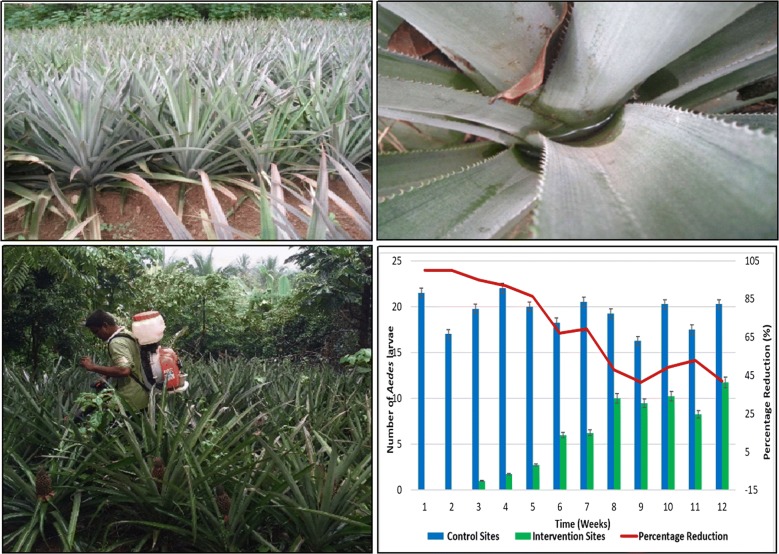

## Background

Dengue is a serious re-emerging mosquito-borne viral disease that affects humans in tropical and subtropical regions of the world. Around 2.5 billion people are at a risk in more than 100 tropical and subtropical countries [1]. Sri Lanka has been engaged in a fight against dengue, since the mid-1960s, which has now developed into a regular epidemic. However, the year 2017 started with an exceptionally high number of dengue cases, which shot up to an outbreak in May–June 2017, creating the largest dengue outbreak experienced by the country reporting 186,101 suspected dengue cases [2]. According to the Epidemiological Unit of Sri Lanka, more than 30,000 cases have been reported every year since 2012, and approximately half of these cases were reported from the Western Province, including Colombo and Gampaha districts [2].

*Aedes aegypti* is considered as the primary vector supported by *Ae. albopictus* as the secondary vector in Sri Lanka [3, 4]. Females of *Aedes* mosquitoes have adapted to oviposit in a wide range of natural breeding habitats. Leaf axils of economically valuable crops and plants such as pineapple, banana, and other ornamental plants are some of the best examples. A preliminary study conducted in Sri Lanka has indicated that the leaf axils of pineapple plants contribute mostly to the breeding of the dengue vector mosquitoes by providing suitable breeding habitats when compared to other plants [5, 6]. At the same time, relevant health institutions have made continuous complaints regarding reported cases of dengue from dwellings located in the proximity of pineapple plantations.

There is a high demand for Sri Lankan pineapple at both local and international markets in terms of its pleasant taste and flavor. In Sri Lanka, there is a huge potential for growing pineapple, because of the ideal agroclimatic conditions [7]. About 90% of the pineapple in the country is grown within the Kurunegala, Puttalam, Gampaha and Colombo districts. Of the total pineapple production, 70% is produced from the Kurunegala and Gampaha districts [8]. The Gampaha District has an ideal climatic and ecological environment for the cultivation of pineapple. Therefore, pineapple plantation growers increase production by expanding cultivation based on the demand in the local and foreign markets.

There is no proper method defined to control mosquito breeding in leaf axils. Therefore, people used to destroy plants containing leaf axils especially during the high epidemics of dengue, which is also encouraged by the control programmes. Being an agricultural country, the removal or destruction of an economically important crop such as pineapple creates controversy and economic damage to the entire country. Therefore, it is important to introduce appropriate control activities to reduce vector densities associated with pineapple plantations. The effectiveness of the application of chemicals such as temephos is questionable due to developing resistance and limited residual effect [9, 10]. Therefore, new chemicals need to be tested to replace temephos in case of the development of resistance.

Insect growth regulators (IGRs) are considered as a novel group of insecticides that can be effectively used to control mosquitoes. The IGRs, in general, exhibit a good margin of safety to most of the non-target biota, thus offering some advantages in mosquito control programmes [11]. Many IGR compounds and products have been evaluated for larvicidal activity against various mosquito species belonging to the genera *Culex* [12, 13], *Aedes* [14] and *Anopheles* [15, 16].

Novaluron is an IGR of the benzoyl urea group, which inhibits the normal development of mosquitoes by inhibiting chitin synthesis and thereby reducing the population density. It inhibits 95% of mosquito emergence and the residual effect lasts nearly two months after application, depending on the type of breeding site [17]. According to the World Health Organization (WHO), novaluron has a high margin of safety for mammals [18]. Therefore, the present study was carried out to evaluate the efficacy of novaluron against immature stages of dengue vectors under laboratory and field settings.

## Methods

### Experimental material

An EC10 (emulsifiable concentrate containing 10% active ingredient) formulation of novaluron was obtained from A. Burse Co. Ltd, Sri Lanka. This material is produced by Makhteshim Chemical Works Ltd, Israel. The larvicidal property of novaluron has not been evaluated previously to control of dengue vectors in Sri Lanka.

### Determination of the discriminative dosage of novaluron

The minimum dosage that provides the highest mortality was determined according to the WHO guidelines for laboratory and field testing of mosquito larvicides [18]. Ten different concentrations of novaluron were prepared in larval rearing trays (25 × 25 × 7 cm^3^) by adding 0.005 ml (2 ppm), 0.01 ml (4 ppm), 0.025 ml (10 ppm), 0.05 ml (20 ppm), 0.06 ml (24 ppm), 0.125 ml (50 ppm), 0.25 ml (100 ppm), 0.5 ml (200 ppm), 0.75 ml (300 ppm) and 1.0 ml (400 ppm) of the manufacturer’s formulation of novaluron into 250 ml of de-chlorinated water. A batch of 25 third-instar *Ae. aegypti* larvae were introduced to each experimental setup described above, which were obtained from the F1 progeny of the laboratory-reared colony maintained at the Department of Parasitology, Faculty of Medicine, University of Kelaniya, Sri Lanka. The mortality rate after 1 h and 24 h exposure periods were recorded.

If mosquito mortality in the control exceeded 10%, the corrected mortality was calculated using Abbott’s formula [18]. The tests were rejected if the corrected mortality in the control exceeded 10%. Where the control mortality exceeded 10%, the tests were rejected. The experimental set-up was repeated four times with control trials.

### Selection of study area and study setup

The Gampaha District of Sri Lanka is located in the Western Province. The district extends over 1387 km^2^ and has a population density of approximately 1800 persons per km^2^, which is the second-highest populated district in Sri Lanka. The average annual temperature is 27.3 °C with an average annual rainfall of 2398 mm. Pineapple is one of the major cultivated crops in the Gampaha District of Sri Lanka [7]. The presence of leaf axils that increase the vector receptivity is considered to be the main reason for elevated dengue transmissions around pineapple plantations. Therefore, the selection of the study site was based on the availability of interested breeding habitat. Hence, the Meerigama (7° 4′ 30.46″ N, 80° 34′ 32.28″ E) Medical Officer of Health (MOH) area was selected for the present study based on the high land coverage of pineapple plantations maintained for commercial interests and the history of reported dengue cases.

### Determination of the IGR efficacy through entomological surveys

#### Baseline survey

**Fig. 1:**
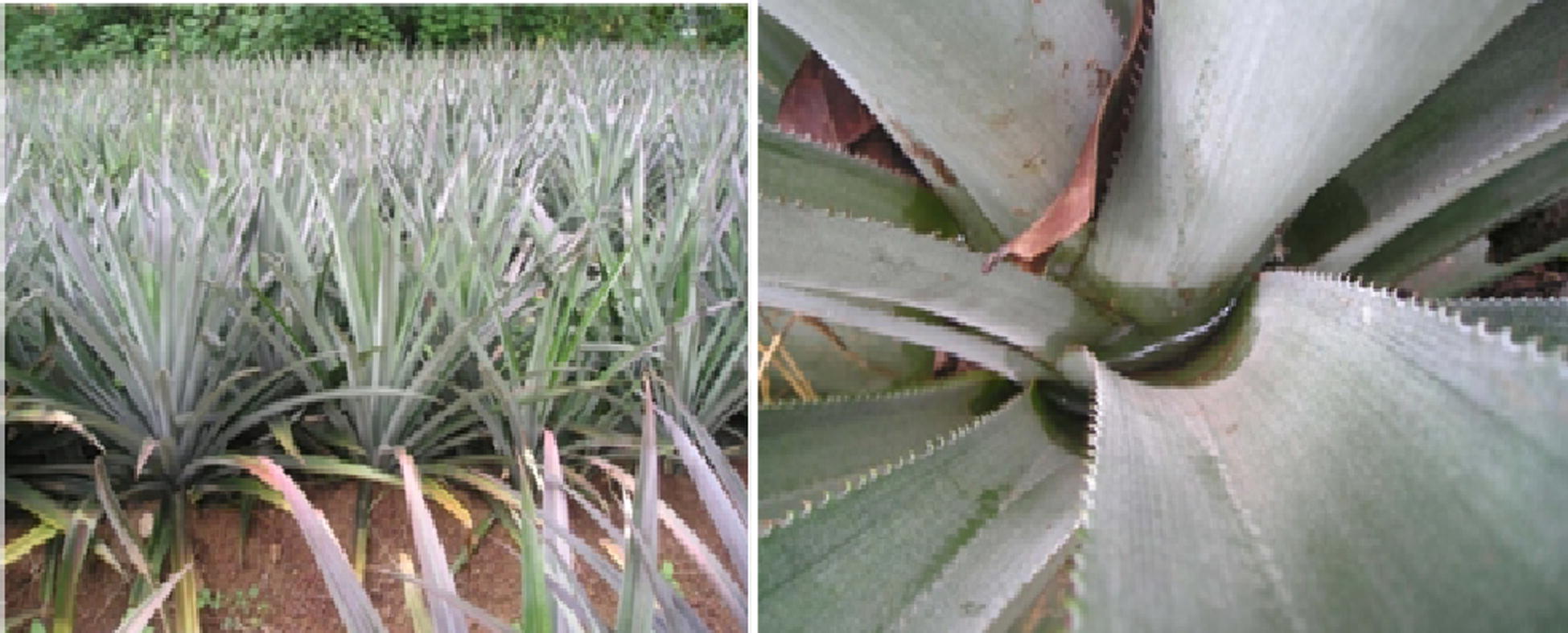
Selected pineapple plantations indicating the accumulation of water in leaf axils

Three pineapple plantations (each covering approximately 2–3 acres) as test and control sites were selected. Control sites were identified with a distance of 750 m away from the test sites. Pineapple plantations of the same age with similar neighbourhoods were selected. Fifteen plots of 10 × 10 m were demarcated with a 200 m distance between any two plots (Fig. 1). Thirty plants in each plot were selected for the survey and a total of 450 pineapple plants (30 plants × 15 plots) were screened for the presence of immature stages of *Aedes* mosquitoes in each of control (3 sites) and test (3 sites) sites during October to December 2018 before the intervention trial. The major intention of the baseline survey was to verify that there is no significant variation of larval densities of *Aedes* among control and intervention sites (within the intervention period) so that any reduction of larval density in intervention sites could be accounted for the IGR activity.

#### IGR application

**Fig. 2:**
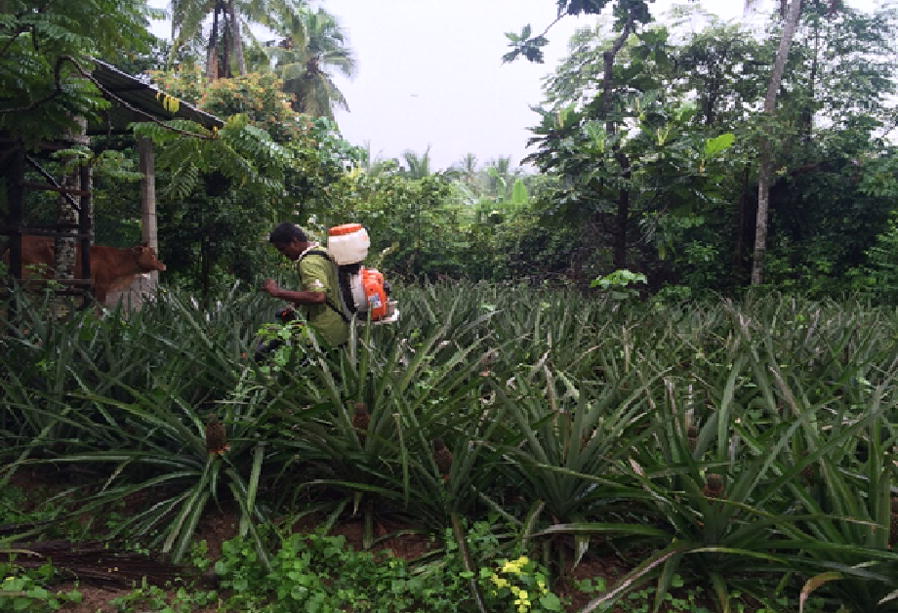
Spraying of novaluron IGR to a selected test pineapple plantation

The required concentration for field application was prepared in a compression spray tank (8 l). The mixture was sprayed to the selected pineapple plants (*n* = 1350) individually in 3 selected test sites (450 plantations each) for 5–10 s through a cone nozzle that reached up to 1 m (Fig. 2). In the present study, IGR was applied at a concentration of 0.05 ml of novaluron 100 g/l in 250 ml of water (20 ppm).

#### Entomological monitoring for efficacy assessment

The immature stages in leaf axils were collected at weekly intervals from January to March 2019 up to 12 weeks after introducing the chemical. The intervention trials with IGR were conducted from January to March 2019. This period corresponds to the latter part of the North-East monsoon and first inter-monsoon in Sri Lanka, with moderate and limited rainfall intensities, respectively. This specific period was selected for the intervention to avoid flushing off the chemical or evaporation by UV in the dry season.

Similarly, entomological surveys were also conducted at selected control sites weekly for 12 weeks. The reduction of the density of immature mosquito stages was interpreted as the percentage reduction of *Aedes* larvae among the control and intervention sites.

### Statistical analysis

The minimum effective concentration of novaluron under laboratory conditions was determined based on the percentage mortality of *Ae. aegypti* larvae. Initially, the percentage mortality rates were calculated for different concentrations of novaluron at 1h and 24 h observation periods as a percentage of the number of dead larvae out of the total number of larvae introduced. General linear model (GLM) followed by Tukey’s *post-hoc* pairwise comparison tests were used to investigate the difference in mortality rates of *Ae. aegypti* larvae at different concentrations of novaluron. Probit analysis was performed to calculate LD_50_, LD_95_ and LD_99_ values of novaluron for *Ae. aegypti* larvae at 1 h and 24 h observation periods.

For field efficacy evaluations, the percentage reduction of the *Aedes* larvae in treatment sites compared to control sites was calculated weekly based on the formula recommended by Mulla et al. [14] and Cetin et al. [19]. This equation evaluates the changes in larval densities caused by the intervention (IGR) while respecting natural changes in mosquito larval populations that may occur simultaneously in both control and intervention sites:

$$ {\text{Percentage}}\;{\text{reduction}} = 100 - \left[ {{{\left( {{\text{C}}_{1} \times {\text{T}}_{2} } \right)} \mathord{\left/ {\vphantom {{\left( {{\text{C}}_{1} \times {\text{T}}_{2} } \right)} {{\text{C}}_{2} \times {\text{T}}_{1} }}} \right. \kern-0pt} {{\text{C}}_{2} \times {\text{T}}_{1} }} \times 100} \right] $$

where C_1_ is the mean number of *Aedes* larvae in control sites during pre-treatment, C_2_ is the mean *Aedes* larval count in control sites during treatment, T_1_ is the mean larval count in intervention sites during pre-treatment (baseline) period, and T_2_ refers to the *Aedes* larval count in intervention sites during the treatment period.

The GLM followed by Tukey’s *post-hoc* comparison was used to evaluate the significance in *Aedes* larval reduction by IGR at a weekly level. In addition, the Chi-square test of independence was used to evaluate the significance between immature stages of *Aedes* larvae among control and intervention sites, prior application of IGR (October to December 2018). Subsequently, the numbers of *Aedes* larvae found in intervention and control sites within the intervention period (January to March 2019) were also compared using the Chi-square test of independence.

## Results

### Larvicidal potential under laboratory conditions

The percentage mortality of *Ae. aegypti* larvae treated with different concentrations of novaluron are shown in Table 1. The 100% mortality was observed at 300 ppm (0.75 ml) concentration of novaluron after a 1 h observation period. The concentration of 20 ppm (0.05 ml) indicated the 100% mortality within 24 h as the lowest treatment intensity. The statistics of the GLM indicated that variations in the percentage mortality rates of *Ae. aegypti* were significant in terms of novaluron concentrations at 1 h (*F*_(9, 30)_ = 198.17, *P *< 0.001) and 24 h observation periods (*F*_(9, 30)_ = 231.88, *P *< 0.001).

**Table 1:** Mean percentage mortality of *Ae. aegypti* at different novaluron concentrations (ppm)

Volume of novaluron (ml)	Novaluron concentration (ppm)	Percentage mortality of *Ae. aegypti*	
		1 hour	24 hours
0.005	2	0 ± 0^a^	43.00 ± 1.08^a^
0.010	4	0 ± 0^a^	59.50 ± 2.25^b^
0.025	10	0 ± 0^a^	86.75 ± 3.40^c^
0.050	20	0 ± 0^a^	100 ± 0.75^d^
0.060	24	0.50 ± 0.28^b^	100 ± 0^d^
0.125	50	11.25 ± 1.10^c^	100 ± 0^d^
0.250	100	49.00 ± 2.48^c^	100 ± 0^d^
0.500	200	96.25 ± 1.93^d^	100 ± 0^d^
0.750	300	100 ± 0^d^	100 ± 0^d^
1.000	400	100 ± 0^d^	100 ± 0^d^

*Notes*: Values are means ± standard errors (SE). Different superscript letters in a row show significant differences (*P *< 0.05) as suggested by the general linear model followed by Tukey’s pairwise comparison at a 95% level of confidence

**Fig. 3:**
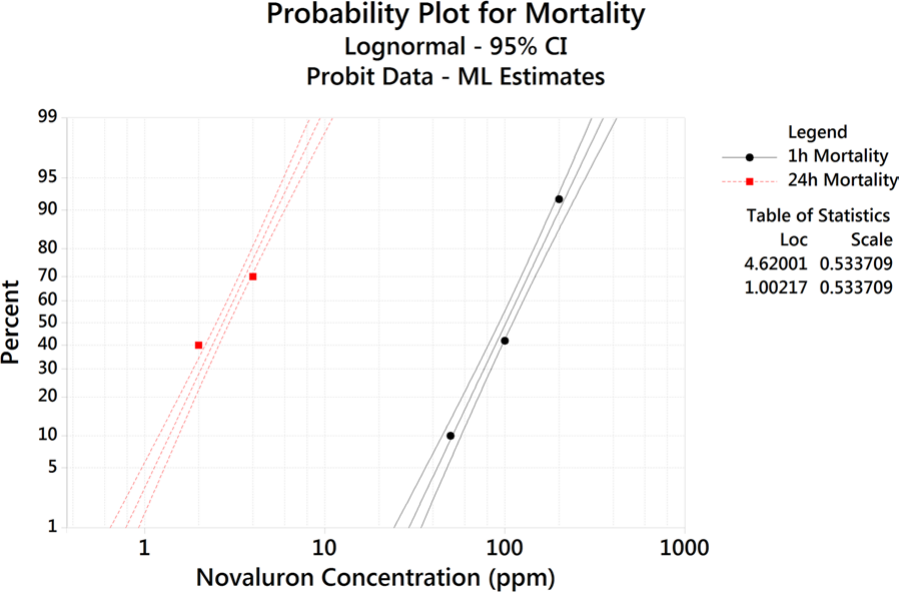
Probability plot for 1 hour and 24 hour mortality of *Ae. aegypti* larvae exposed to different concentrations of novaluron

The Probit analysis (based on a lognormal distribution) indicated a relatively steeper slope at 24 h mortality than at 1 h mortality (Fig. 3). The difference in the slopes was observed as statistically significant (*χ*^2^ = 7.67, *df* = 1, *P* = 0.006). Based on 24 h mortality of novaluron, the calculated lethal concentration for 50% (LC_50_), 95% (LC_95_) and 99% (LC_99_) elimination of *Ae. aegypti* larvae were 2.72 ± 0.11 ppm, 6.56 ± 0.37 ppm and 9.43 ± 0.69 ppm, respectively (Table 2).

**Table 2:** Lethal concentration values for novaluron at 1 hour and 24 hours for *Ae. aegypti*

Lethal concentration	Mortality of *Ae. aegypti*	
	1-hour mortality (ppm)	24-hour mortality (ppm)
LC_50_	101.49 ± 4.45	2.72 ± 0.11
LC_95_	244.18 ± 15.67	6.55 ± 0.37
LC_99_	351.29 ± 28.37	9.43 ± 0.69

*Note*: Values are means ± standard errors (SE)

### Larvicidal potential under field conditions

**Fig. 4:**
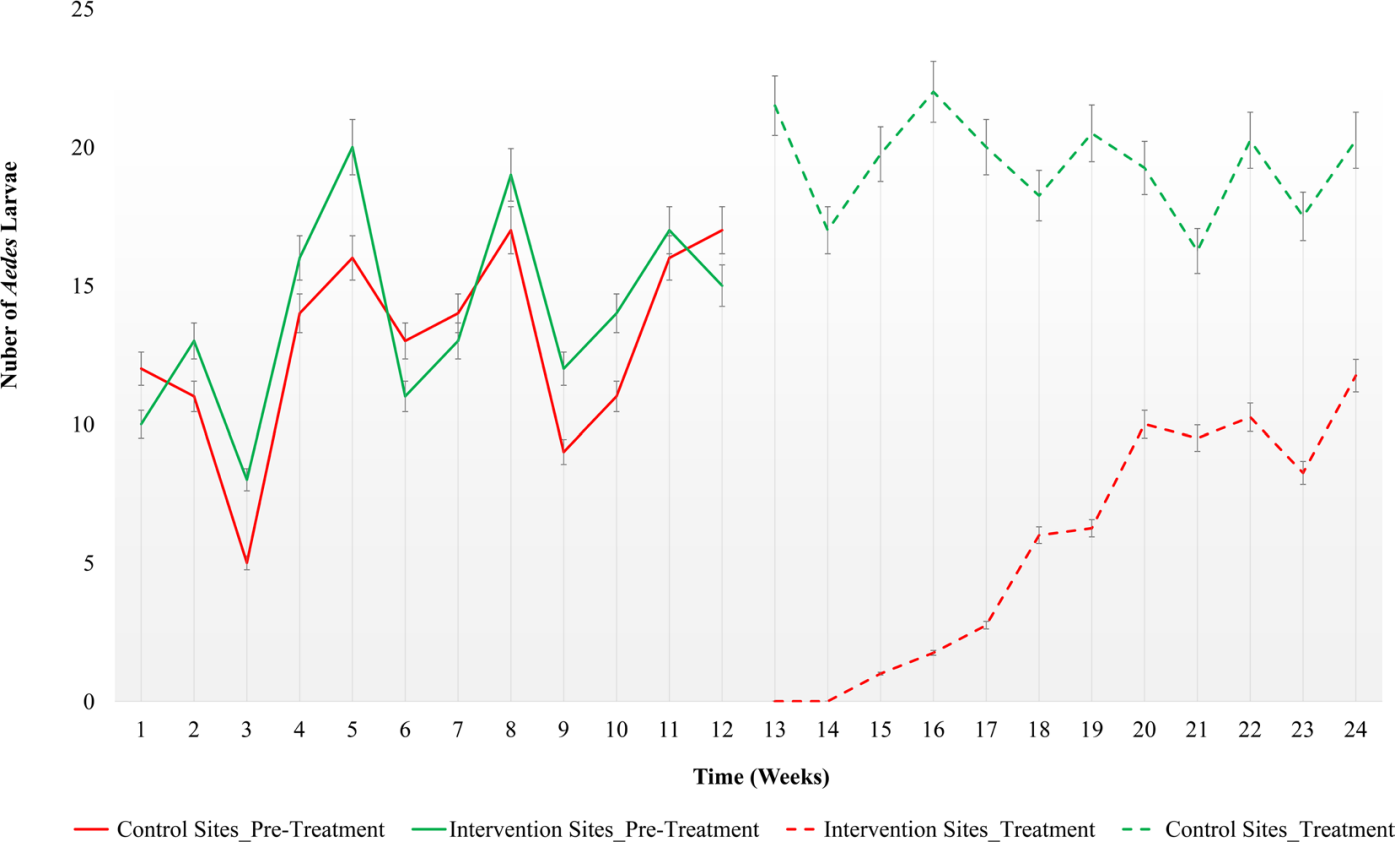
Temporal variation of the mean number of *Aedes* larvae in control and intervention sites in pre-treatment (baseline) and treatment (after application of novaluron 20 ppm) periods

The temporal variations of the mean number of *Aedes* larvae identified from the baseline survey and during the treatment period in control and intervention sites are indicated in Fig. 4. Based on the Chi-square statistics, the mean number of *Aedes* immature stages in intervention and control sites did not show any significant difference (*χ*^2^ = 17.108, *df* = 11, *P* = 0.105), during the baseline period. The mean number of *Aedes* larvae present at control sites before and after the application of novaluron (20 ppm), did not show any significant variations across the observation period of 12 weeks (*χ*^2^ = 7.27, *df* = 11, *P* = 0.803). Meanwhile, a significant variation (*χ*^2^ = 34.82, *df* = 11, *P *< 0.0001) in the mean number of *Aedes* larvae was reported from the intervention sites during the intervention phase in comparison to the baseline period. Further, the variations in the number of *Aedes* larvae present at control and intervention sites were significantly different throughout the treatment period (*χ*^2^ = 128.29, *df* = 11, *P *< 0.001).

Table 3 indicates the mean percentage reduction of *Aedes* larvae caused by the field application of IGR. It was noted that the field application of IGR at 20 ppm is sufficient for 100% elimination of *Aedes* immature stages up to 2 weeks. Further, the reduction percentage remained at > 50% until the 8th week (53.5%) from the initial application. The lowest reduction was observed as 34.2% after the 12th week of application (Table 3). The statistics of the GLM denoted that the percentage reduction of *Aedes* larval count differed significantly (*F*_(11, 24)_ = 25.89, *P *< 0.001) throughout the observation period of 12 weeks. Based on the *post-hoc* analysis, it was found that four sub-clusters of observation weeks were found: (i) 1 to 5 weeks; (ii) 6 to 7 weeks; (iii) 8 to 11 weeks; and (iv) 12 weeks (Table 3).

**Table 3:** Mean percentage reduction of *Aedes* larvae caused by the field application of IGR

Week of observation	Percentage reduction
1	100 ± 0.0^a^
2	100 ± 0.34^a^
3	96.8 ± 2.78^a^
4	93.0 ± 2.82^a^
5	89.0 ± 3.89^a^
6	61.1 ± 5.41^b^
7	67.2 ± 4.87^b^
8	53.5 ± 5.51^c^
9	48.7 ± 5.09^c^
10	45.6 ± 4.02^c^
11	41.9 ± 3.05^c^
12	34.2 ± 4.01^d^

*Notes*: Values are means ± standard errors (SE). Different superscript letters show significant differences (*P *< 0.05) as suggested by the general linear model followed by Tukey’s pairwise comparison at a 95% level of confidence

## Discussion

Dengue vectors have been known to breed in several kinds of containers [20] and their presence has also been reported in different types of leaf axils such as banana, *Alocasia*, *Colocasia*, *Dracaena*, *Pandanus*, pineapple and other bromeliads [21, 22]. The availability of water-filled leaf axils is influenced greatly by agriculture and those of pineapple are a principal habitat. The frequency of rainfall is a major determinant of the water content in those axils. Although the volume of water in each axil is usually small, it is adequate for mosquito breeding. In addition, a higher percentage of these axils will harbor mosquito larvae during rainy seasons [21].

Health authorities in Sri Lanka currently use temephos (Abate) to control the dengue vector population in pineapple plantations. The WHO has recommended the dosage of Abate at 1% SG to eliminate the larvae population. A solution of 1 g per 10 liters of water gives effective control up to a period of 2.5 to 5 months, with an average of 3 months [23]. A study carried out in Mexico showed that the effectiveness of temephos against vectors of dengue significantly declined from day 7 after application [24]. Development of resistance to temephos in *Aedes* mosquitoes has been reported in many countries including Indonesia [25], Brazil [26], Cuba [27], El Salvador [28] Argentina [29], Bolivia [30], Venezuela [31], Peru [32] and Thailand [27]. Therefore, with limitations such as residual effect, developing resistance and toxicity to mammals, insects or other organisms, an alternative should be available next in line.

The present investigation demonstrates that the potential use novaluron as a larvicide for the control of *Aedes* mosquitoes that breed in leaf axils of plants such as in pineapple plantations. Novaluron is an IGR of the benzoyl urea family which acts as a chitin synthesis inhibitor in insects. In general, it has low acute, subacute and chronic toxicity compared to other larvicides used for insect control with no evidence of carcinogenicity, mutagenicity or teratogenicity [18].

The WHO has recommended the use of novaluron as a mosquito larvicide for application in temporary mosquito habitats, polluted waters and non-drinking water-storage [33]. In addition, considering the toxicological aspects of the novaluron, the WHO has recommended novaluron to control the breeding of disease-carrying mosquitoes in drinking-water containers at a dosage not exceeding 0.05 mg/l [18]. Some countries like Canada and the USA have also advocated the use of novaluron as a larvicide to control *Ae. aegypti* and *Ae. albopictus* in breeding sites as a control measure of Zika virus [18].

In Sri Lanka, novaluron 10% EC has been evaluated for efficacy and residual activity in riverine pools in Galewela and gem pits in Elahera which is a common habitat of *Anopheles culicifacies* and *An. subpictus* [34]. This study has indicated that this IGR has a high level of activity against *Anopheles* mosquitoes with 0.01 ml/l dosage. The residual effect lasts for more than 8 weeks in riverine pools and < 12 weeks in gem pits. However, no attempt has been made so far to evaluate the uses for *Aedes* vector control in Sri Lanka or application in natural breeding habitats of *Aedes* vectors such as leaf axils in anywhere in other countries.

According to some studies, there is evidence that novaluron inhibits adult emergence and induce mortality on mosquito larvae depending on the application dose [35, 36]. It has been reported that 10–20 μg of the active ingredient of novaluron added to 200 liters of water in storage jars provides excellent control of the *Ae. aegypti* larvae. The present study reveals that 100% mortality after a 24 hour exposure period was observed even at a low concentration of novaluron (0.05 ml of novaluron 100 g/l in 250 ml of water). However, due to practical issues in field application and properties of the chemical (low vapor pressure and low water solubility) [18], working concentration for the field-based application was adjusted to 20 ppm (0.05 ml of novaluron 100 g/l in 250 ml of water).

The field-based investigation indicated that with the application of IGR, 100% mortality was achieved 2 weeks after post-application and more than 50% of the reduction was observed until the 8th week after introduction. Fontoura et al. [35] have expressed that novaluron inhibited 70% of adult emergence in *Ae. aegypti* and residual effect of the chemical lasted 5–6 weeks in outdoor conditions. Further, Farnesi et al. 2012 [36] stated that novaluron can effectively reduce the chitin content of *Ae. aegypti* larvae. Several studies conducted for *Culex quinquefasciatus* have highlighted the variation among residual activity at different breeding habitats such as cesspits (11–13 days), drains (8–17 days), unused wells (33–69 days), small buckets (10 weeks) and polluted water in urban areas (3–7 weeks). This may be due to the environmental depletion of the active ingredient in the chemical caused by ultraviolet light and the organic pollution level in the water of the habitat [17, 37]. In the present study, we did not estimate the average volume of water in the leaf axils, which would affect the outcome of IGR at the individual plant. Therefore, this can be highlighted as a limitation in the current study. In addition, the baseline and interventional studies were conducted in different months. Therefore, some changes in weather conditions and the occurrence of breeding sites may influence results. The association between rainfall and density of vectors may be confounded by changes in the environment that may affect the mosquito population and the effectiveness of the IGR. However, the baseline survey was mainly aimed at identifying suitable pineapple plantations that would be used for interventional trials. Therefore, the inclusion of test and control sites from the same plantation plots may compensate for the above limitation. Further, the present study did not observe that the remaining immature stages in the leaf axils after invention would emerge as adults, which may be emphasized as a limitation for an IGR based trial. However, the reduction of immature stages compared to test and control sites would reflect the larvicidal effect of the IGR.

The present study examined the residual effect of 12 weeks for the IGR applied in pineapple plantations. As these breeding habitats contain relatively clean water, degradation due to pollutants is negligible. Additionally, novaluron undergoes slow photolysis [18] and the water in leaf axils is not directly exposed to sunlight compared to other breeding habitats mentioned above. Therefore, these properties may facilitate to maintain a durable half-life in the novaluron IGR for a considerable period of time.

Re-treatment of IGR could be suggested between the period of week 9–10 since the percentage mortality remains greater than 50%. Natural degradation of the active ingredients may be recognized as the cause of a decline in the persistence of IGR. In addition, the use of novaluron would be beneficial in vector control since it has the affinity to attract female mosquitoes for oviposition through chemical cues of gravid mosquitoes [38]. Therefore, novaluron could be used as an appropriate candidate in controlling the immature stages of mosquito vectors in breeding habitats.

## Conclusions

The present study emphasizes novaluron (10% EC) would be used effectively to control the breeding of dengue vectors in Sri Lanka at a dose of 20 ppm for leaf axils with a residual effect for 12 weeks and a functional efficacy of 8 weeks. Therefore, re-treatment may be recommended based on the reduction in the efficacy of IGR. Furthermore, this may provide a vital contribution to control dengue vectors in Sri Lanka due to its low toxicity profile (mammals, birds, fish, earthworms and aquatic plants), higher residual effect and no evidence of resistance development. Hence, it is recommended to evaluate the efficacy of novaluron for the control of other breeding habitats of mosquito vectors in Sri Lanka and cross-resistance with other pyrethroids.

## Data Availability

The datasets supporting the conclusions of this article are included within the article.

## Abbreviations

GLM: general linear model (GLM)
IGR: insect growth regulators
LC: lethal concentration
MOH: Medical Officer of Health
SE: standard error
WHO: World Health Organization
